# The effect of combined red, blue, and near-infrared light-emitting diode (LED) photobiomodulation therapy on speed of wound healing after superficial ablative fractional resurfacing

**DOI:** 10.1007/s10103-024-04042-x

**Published:** 2024-03-27

**Authors:** John Soliman, Rachel Elsanadi, Feben Messele, Kristen M. Kelly

**Affiliations:** 1 Department of Dermatology, University of California, Irvine, 118 Medical Surge I, Irvine, CA 92697 USA; 2 Beckman Laser Institute, University of California, Irvine, Irvine, CA USA

**Keywords:** Ablative fractional laser, LED treatment, Light-emitting diode, Wound healing

## Abstract

Objective of the study is to assess the effects of wound healing with a commercially available light emitting diode (LED) photo biomodulation (PBM) device that emits three wavelengths (465, 640 and 880nm) after ablative fractional laser (AFL) treatment to healthy skin on the bilateral inner biceps. We conducted a prospective intraindividual randomized controlled study with 25 volunteers. AFL treatment was performed on healthy skin of the bilateral inner biceps. Subjects applied the LED light device for 30 min to the assigned bicep 3 times a week over 4 weeks, beginning on day 0. Subjects were followed up on days 2, 4, 6, 9, 13, 20 and 27 for treatment with the PBM device, clinical digital photography of the test and control sites, and in-person subject assessment, with follow ups on days 34 and 55 for clinical photography and assessment. Three blinded evaluators were asked to determine which bicep healed faster between day 0 to day 13. Pain, discomfort, and itch were also assessed. The three blinded evaluators chose the treatment arm as the faster healed arm in greater than 50% of the images, although the results were not statistically significant. There was no statistically significant difference between test and control arms in terms of pain, discomfort and itch. In conclusion, PBM therapy has the potential to improve wound healing. In this study, a three wavelength PBM device resulted in some subjects achieving faster healing after AFL but the results were not statistically significant.

## Introduction

Since the 1960’s, photobiomodulation (PBM) therapy has been used in wound healing [1]. This process involves exposing cells and tissue to low levels of light, as compared to the higher energy densities of traditional photothermolysis based laser therapy. In the last several years, major improvements have been made to light emitting diodes (LEDs), and they have begun to replace lasers as a light source for PBM therapy [2–5].

Studies utilizing PBM therapy from 600 to 1072 nm in vitro and in animal models have demonstrated a positive effect on wound healing [3]. In humans, normal wound or tissue healing is a sequential process with three phases: inflammatory, proliferative, and matrix remodeling [6]. Potential biological effects of PBM have been proposed for all three phases of the wound healing process [7–9]. Various mechanisms have been proposed including metabolic effects of light therapy on tissue repair and faster resolution of the inflammatory process [8]. An in-vitro study by Hawkins et al. demonstrated lower light doses of PBM therapy (2.5 J/cm^2^) led to changes in the respiratory chain in the mitochondria and enhanced DNA and RNA synthesis leading to cell proliferation [10]. PBM has also been shown to induce fibroblast proliferation and collagen synthesis aiding the proliferative stage of healing [8]. PBM therapy can significantly increase the synthesis of fibroblast growth factor (FGF), which contributes to collagen synthesis [8–10]. Hopkins et al. demonstrated PBM therapy treated wounds showed earlier contraction and subsequent healing. [11]. Other studies demonstrated that PBM therapy can induce mast cell degranulation, which through the release of proinflammatory granules, can assist in collagen synthesis [12]. In addition, PBM therapy has been demonstrated to enhance local blood flow and lymphatic drainage, which can accelerate wound healing by delivering oxygen to the tissues and clearing away waste through the lymphatic system [13].

There may be different effects on wound healing for different wavelengths. Adamskaya et al. demonstrated rats exposed to blue light (470 nm) had faster healing attributed to the effect of blue light on nitric oxide (NO) metabolism—including the release of NO from hemoglobin complexes, with better perfusion of tissues and release of NO from mitochondrial complexes, improving mitochondria recovery [14]. Wang et al. analyzed the effect of four wavelengths (420 nm, 540 nm, 660 nm or 810 nm) at a dose of 3 J/cm^2^ using human adipose-derived stem cells on osteogenic differentiation of stem cells [15]. They found that blue and green light had better impact on the osteoblast differentiation process (mechanism suggested to be due to activation of light-gated calcium channel) compared with red/NIR light [15]. Red/NIR light had greatest impact on stem cell proliferation (through activation of cytochrome c oxidase in mitochondria) [15].

In a study conducted by Trelles et al., the effect of 633 nm PBM therapy was investigated in 10 subjects who had undergone upper eyelid blepharoplasty [16]. Treatment sessions were 20 min each and performed immediately after and 48 h after surgery, and twice during the week following the surgery [16]. Edema, erythema and bruising were assessed by an independent plastic surgeon and pain, was assessed by the subjects [16]. The study found a significant difference between time to complete healing on the half of the face that was treated with LED light versus the untreated half of the face, with a mean of 13.5 ± 0.34 days for the treated side and 26.8 ± 0.49 days for the untreated side [16]. In addition, the study found statistically significant differences in edema, erythema, bruising, and pain scores between the two arms. Mean edema scores were 3.7 compared to 7.6, mean erythema scores were 3.7 compared to 7.6, mean bruising scores were 4.7 compared to 8.5, and mean pain scores were 2.9 compared to 6.9 for treated versus untreated sides respectively [16]. However, at 6 weeks post-blepharoplasty, there was no significant difference in skin quality between the LED treated and untreated sides [16].

Ablative fractional laser (AFL) is used for photo-rejuvenation and treatment of scars [17]. While generally well tolerated, there is a healing phase of several days to a week or more during which patients may experience a sunburn type sensation, oozing, and discomfort. PBM therapy has the potential to improve healing post AFL. Le Duff et al. conducted a study to investigate the effects of different wavelengths on wound healing in humans in a randomized comparative intraindividual study [17]. Each of the ten participants, with skin types I-III, had treatment with placebo, 590, 630 nm or 850 nm PBM therapy to different test spots following AFL (CO_2_: 10,600 nm; Fraxel Re:Pair Solta Medical/Bausch Health companies—handpiece 135 μm, 15 mm, fluence 30 mJ, and density 30%) [17]. The authors did not find a statistically significant difference in erythema or transepidermal water loss at day 3 post-laser between the placebo and PBM treated wounds for any of the tested wavelengths [17].

Studies as described above have assessed the effect of a specific wavelength on wound healing, though there is little research on the effect of a combination of wavelengths. The objective of the current study was to evaluate the effect of a device with a combination of blue (465 nm), red (640 nm and near infrared (880 nm) LED PBM therapy on wound healing after application of low-density AFL to bilateral inner biceps following up for a period of 55 days. Given the three wavelengths act through different mechanisms to improve wound healing, our hypothesis was LED PBM therapy would result in less post treatment pain and faster healing after AFL**.**

## Methods

This study was approved by the UC Irvine Investigational review board. Inclusion criteria were age 18 years or older and exclusion criteria were conditions, topicals, or medications causing photosensitivity, active smoker, history of epilepsy or seizures, current treatment with cortisone or other steroid injections, or malignancy in the treatment area. All patients signed approved consent forms. Patient were randomized to left or right PBM therapy by a randomized left/right generator. On the day of the procedure, photographs were taken and a two-inch by two-inch treatment area was marked with a skin marker. Equal areas on the left and right bilateral inner biceps were marked. The areas were cleaned with chlorhexidine, followed by sterile water and then carefully dried. All present during the treatment wore appropriate laser safe eye goggles. AFL treatment (Sciton Profractional, Palo Alto, CA) was then performed (3 spot; 300 µm; 5.5% density). Air cooling and a smoke evacuator were used during treatment and no other form of anesthesia was used. Subjects were evaluated for pain and discomfort immediately after the treatment using an 11-point visual analogue scale. Clinical digital photography (iPhone 13 Pro) of the test and control sites post induction were recorded. Immediately after photography, the test area was exposed to the LED light devicefor 30 min. The device used simultaneously emits blue: 465 nm ± 22 nm, red: 640 nm ± 25 nm and near infrared 880 nm ± 50 nm light. Treatment parameters were: irradiance 6.5 mW/cm^2^, fluence 11.7 J/cm^2^, Spot Size: 3 × 3 cm^2^. The control side was left untreated. Following treatment with the LED light device, subjects were again evaluated for pain and discomfort for the test and control sites using the same 11-point visual analogue scale.

**Fig. 1:**
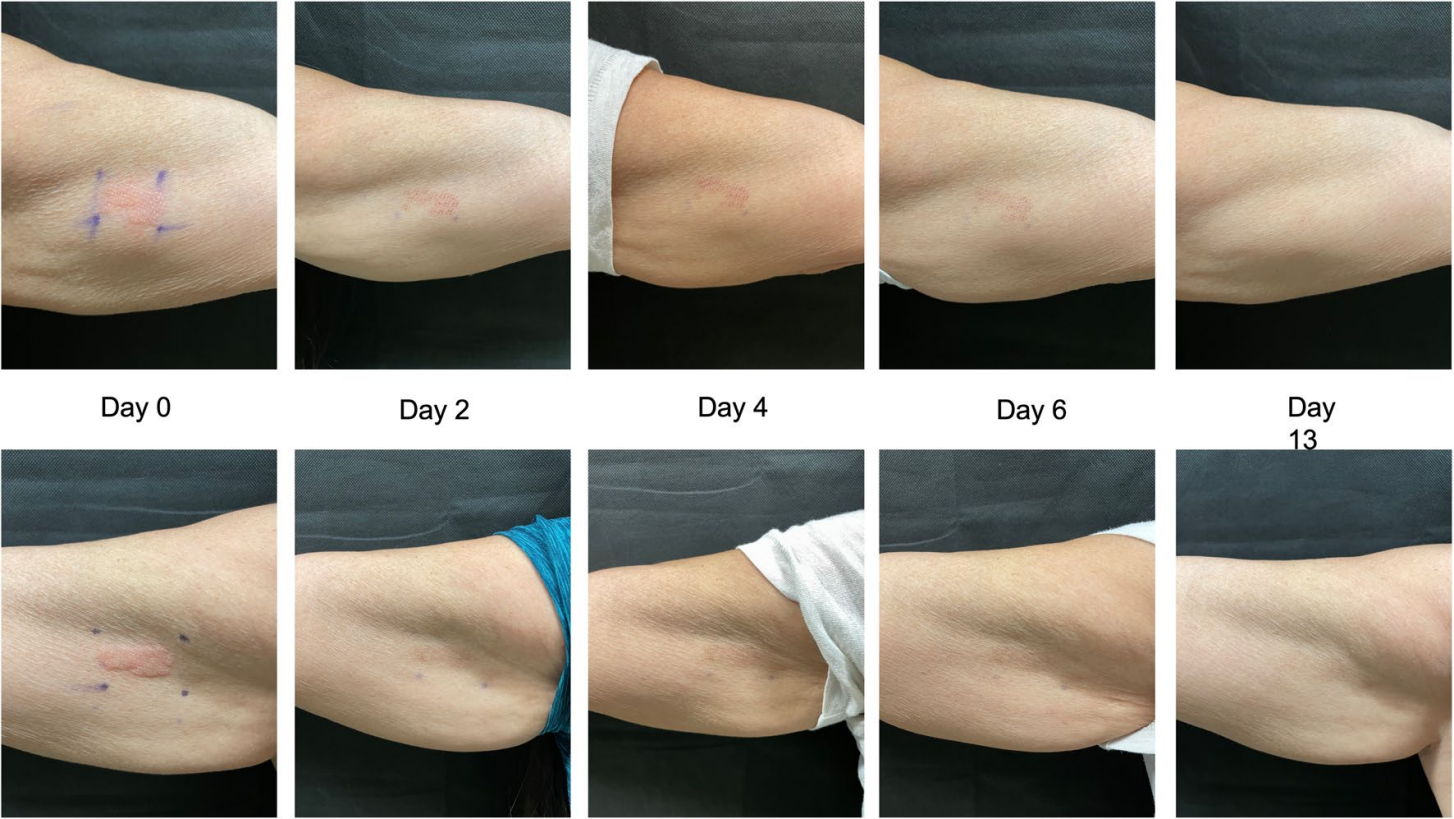
The figure shows the left inner bicep (top set of images) and right inner bicep (bottom set of images) of subject 35 on day 0 immediately after ablative fractional laser (AFL) treatment, day 2, day 4, day 6, and day 13. The right inner bicep was treated with light emitting diode (LED)

**Fig. 2:**
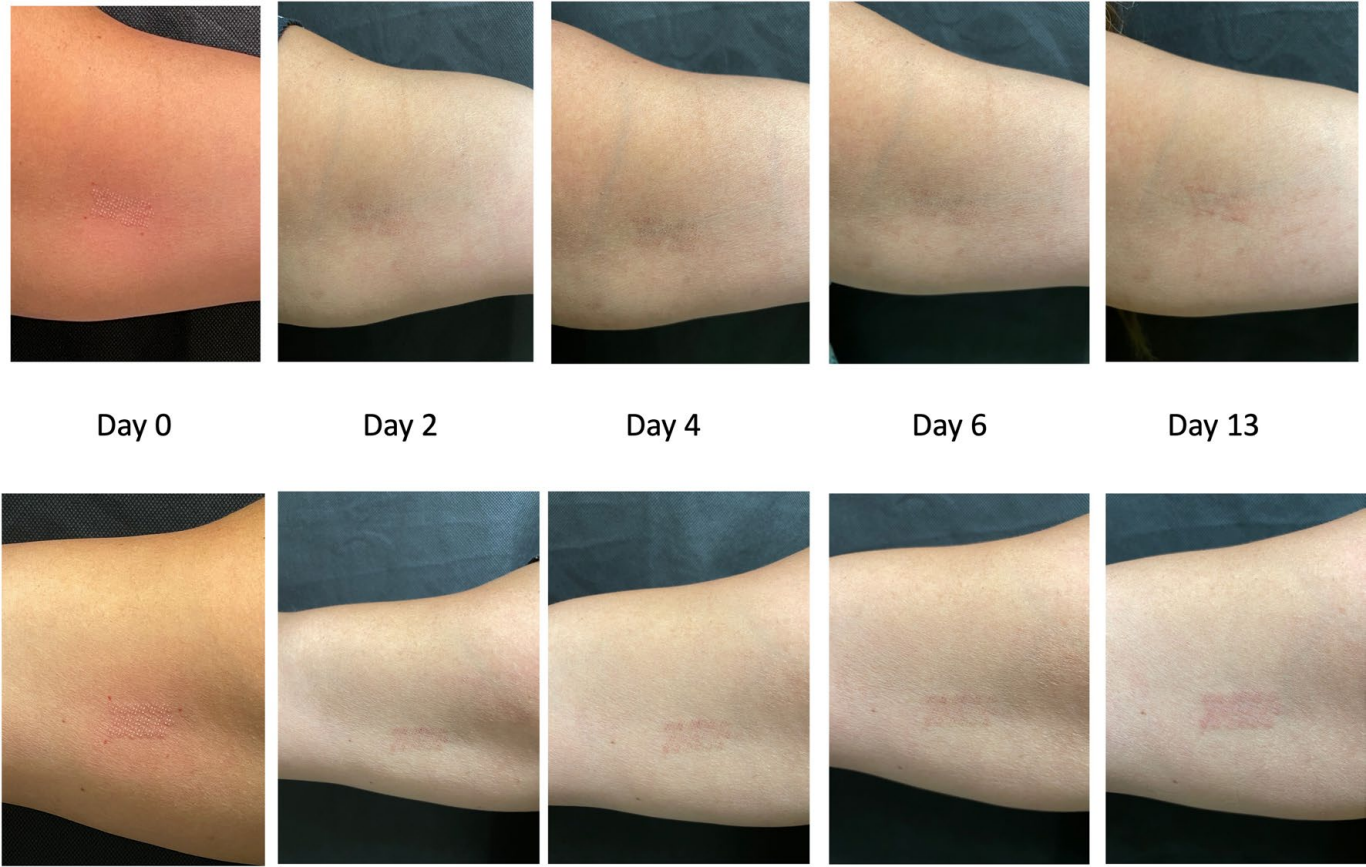
Left inner bicep (top set of images) and right inner bicep (bottom set of images) of subject 10 on day 0 immediately after ablative fractional laser (AFL) treatment, day 2, day 4, day 6, day 13. The right inner bicep was treated with light emitting diode (LED)

Subjects then applied the LED light device for 30 min to the assigned bicep 3 times a week over 4 weeks. In addition to at-home treatments, subjects were also followed up on days 2, 4, 6, 9, 13, 20, and 27 for treatment with the device, clinical digital photography of test and control sites, and treatment site assessment (Figs. 1 and 2). Following the four weeks of treatment, subjects were followed up on days 34 and 55 for clinical photography and assessment.

A photograph of each arm was taken at every follow up appointment. The images for each patient taken on days 0, 2, 4, 6, 9, and 13 were assessed by three blinded physician evaluators who were not previously involved in the study and specialized in either dermatology or plastic surgery. The evaluators were asked to identify which arm healed faster over the course of day 0 to day 13. We chose to evaluate at day 13, because the majority of healing was complete at that time point.

## Results

**Fig. 3:**
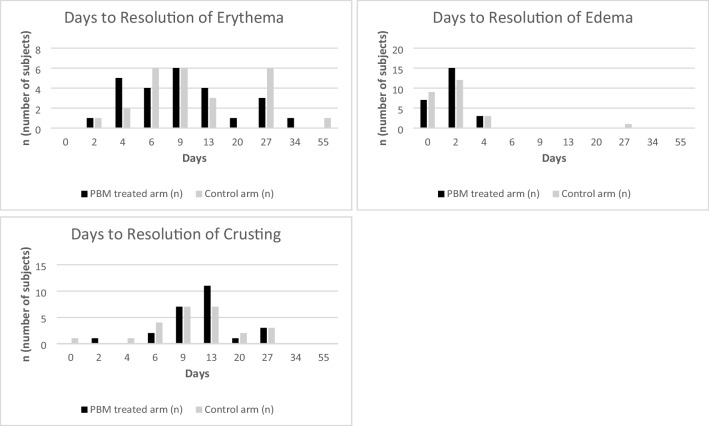
Days to resolution of Erythema, Edema, Crusting, followed up to day 55 in both groups; PBM treated arm and control arm

A total of 25 subjects received laser treatment on day 0 and were subsequently followed to day 55 post-treatment, with photographs evaluated by blinded evaluators up to day 13. A small number of subjects had residual erythema or pigmentary change after this time point. In the PBM therapy treated arms, erythema (Fig. 3) was completely resolved by day 27 for all but one subject, which cleared by day 34. In the untreated arms, erythema was resolved also by day 27 except for one subject with clearance by day 55. For both treated and untreated arms, edema (Fig. 3) was resolved by day 4 except for one subject in the untreated arm, resolving by day 27. Of note, some of the early subjects put significant pressure on the skin while holding the LED-light device during PBM therapy contributing to short term significant erythema and edema that rapidly resolved within hours. Instructions were adjusted so patients held the device less tightly, which resolved these short-term effects.

For both treated and untreated arms, crusting (Fig. 3) was resolved in most patients by day 13 except for in three subjects in each treated and untreated arm, resolving by day 27. Two subjects developed mild post-inflammatory hyperpigmentation (PIH), one subject developed PIH in both the treated and untreated arms and this resolved by day 9. The other subject exhibited mild hyperpigmentation in the arm not treated with PBM therapy and not completely resolved on day 55.

As noted above, three blinded independent evaluators, not previously involved in the study, reviewed post-treatment photographs (Figs. 1 and 2) up to day 13. The first evaluator chose the treated arm as the faster healed arm for 17/25 patients. The second evaluator chose the treated arm as the faster healed arm for 14/25 patients. The third evaluator chose the treated arm as the faster healed harm for 14/25 patients.

Subjects were also subjectively evaluated on pain, discomfort, and itch before and after treatment with the LED light device at each follow up appointment. The mean decrease in pain scores in the treated arms at 0 and 30 min following LED therapy on day 0 was 0.6 compared to 0.48 in the untreated arms. The mean decrease in discomfort scores in the treated arms at 0 and 30 min following LED therapy on day 0 was 1.12 compared to 1.08 in the untreated arms. The mean differences in pain and discomfort scores between the two were not statistically significant.

Four days post-laser, 23/25 subjects were pain and discomfort-free on both treated and untreated arms, while the two remaining subjects’ pain and discomfort on the PBM treated arm resolved by day 6. Three subjects reported itch (two with score of 1, another with a score of 2, on an 11-point scale) on the PBM arms on days 9 and 13, which all resolved by day 13.

## Discussion

The use of LED light to facilitate wound healing has increased worldwide. LED light has little risk of harming patients and is painless, accessible, and feasible for many patients, so well controlled translational studies are needed to determine best uses. We used a combination of blue (465 nm), red (640 nm), and near infrared (880 nm) LED light in a commercially available device as opposed to previous studies in the literature which mostly investigated a single wavelength; this device delivers the three wavelengths simultaneously and there was no option to select a single wavelength. The three blinded independent evaluators chose the treatment arm as the faster healed arm in greater than 50% of the images, although the results were not statistically significant. Having a greater sample size could have possibly yielded statistically significant differences.

Despite the difference in our methods from the study by Le Druff et al., we also found PBM therapy did not produce a statistically significant improvement in wound healing for superficial ablative resurfacing [17]. Le Duff et al., utilized PBM therapy and looked at single wavelength exposures at 590, 630 or 850 nm following AFL to the bilateral arms [17]. For the Le Duff et al. study, the wounds were subjected to different times and different wavelengths, with the longest treatment being 15 min, while for our study, the treatment with PBM lasted 30 min [17]. We also compared the difference in wound healing until day 13 post-laser as opposed to day 3 in the study by Le Duff et al. [17] Our study also had deeper laser penetration [17]. Similarly to Le Druff et al., our study used a lower light power density and the inner arm was the treated area [17]. On the contrary, Trelles et al. found statistically significant resolution in healing using “red LED light phototherapy” after blepharoplasty and laser ablative resurfacing [16]. There are some notable differences between our study and Trelles et al. Trelles et al. used a single wavelength to treat the face with a higher LED light power density (80 and 96 J/cm^2^ over 20 min) [16].

In the study by Trelles et al., the higher severity of the blepharoplasty procedure may have provided a greater difference observed between test and control compared to the procedure in our study, in which we went 300 microns deep into the skin with a fractionated device [16]. Le Duff et al. similarly performed AFL to a depth of only 135 μm [17]. The body part treated may also have affected the results, as the bilateral arms may heal differently as compared to face [17].

In summary, our study of 25 subjects did not yield a statistically significant difference. While there is substantial evidence in the literature demonstrating LED light benefit for wound healing in animal and in vitro studies, clinical translational studies are necessary. There is a need for further studies to investigate the effects of LED PBM therapy on wound healing. Effects may be subtle and large study populations may be required.
